# Manganese toxicity impairs soil nitrification and sugarcane nitrogen utilization by inhibiting rhizosphere ammonia-oxidizing microorganisms

**DOI:** 10.3389/fpls.2025.1674405

**Published:** 2025-12-01

**Authors:** Hanyu Zhu, Junchen Pan, Yuqi Qin, Yanyan Wei, Xiaofeng Li, Shu Yang, Baoshan Chen, Taolian Zhong, Tong Zhang, Xinlian Tang

**Affiliations:** 1 State Key Laboratory for Conservation and Utilization of Subtropical Agro Bioresources, Guangxi Key Laboratory for Sugarcane Biology, College of Agriculture, Guangxi University, Nanning, China; 2 Guangxi South Subtropical Agricultural Sciences Research Institute, Chongzuo, China; 3 Agricultural Technology Extension Station of Yinhai District, Beihai, China

**Keywords:** manganese toxicity, sugarcane, ammonia-oxidizing microorganisms, soil nitrification, nitrogen uptake and utilization

## Abstract

**Introduction:**

Sugarcane (*Saccharum spp*.) is an economically important crop cultivated primarily for sugar and bioethanol production. In southern China, sugarcane grown in acidic soils often exhibits severe leaf chlorosis owing to excessive soil manganese (Mn) levels. However, the mechanisms by which Mn toxicity disrupts soil nitrogen (N) cycling, particularly the roles of ammonia-oxidizing bacteria (AOB) and archaea (AOA) in regulating nitrification and N availability in sugarcane, remain unexplored.

**Methods:**

To address this gap, we conducted laboratory soil incubation experiments and greenhouse pot trials using four treatments consisting of combinations of two N levels (N1:0.14 g·kg^-1^; N2:0.28 g·kg^-1^) and two Mn levels (−Mn: 0 mg·kg^-1^; +Mn: 328 mg·kg^-1^, simulated using anhydrous Mn sulfate), along with a blank control (CK). Key measurements included rhizosphere soil physicochemical properties, AOB/AOA community structure, nitrification potential, and sugarcane N uptake efficiency.

**Results:**

Results showed that Mn toxicity significantly reduced soil pH and nitrification potential by 75.9–78.0% compared to non-Mn treatments, and AOB amoA gene abundance by 44.9–46.5%, while altering AOB/AOA community composition. Redundancy analysis (RDA) identified soil organic carbon, total nitrogen, and ammonium nitrogen as the primary drivers of AOB community shifts, whereas exchangeable Mn, ammonium nitrogen, and pH dominated AOA community changes. Correlation analysis confirmed that nitrification potential and AOB amoA abundance were strongly positively linked to sugarcane N accumulation and uptake efficiency, which decreased by 47.3–53.4% under Mn toxicity due to reduced nitrate availability.

**Discussion:**

These findings indicate that Mn toxicity impairs sugarcane N utilization by disrupting ammonia-oxidizing microbial communities and suppressing nitrification, thereby providing insights for optimizing N management strategies in Mn-contaminated acidic soils.

## 1 Introduction

Manganese (Mn) is an essential micronutrient involved in various physiological processes concerning plant growth and development (Shao et al., 2017). During photosynthesis, Mn serves as an essential component of the oxygen-evolving complex in photosystem II, donates electrons for photochemical reactions, regulates the photosynthetic electron transport chain, affects ATP and NADPH production, and ultimately influences carbon assimilation (Livorness et al., 2017). However, excessive Mn accumulation is toxic to plants (Li et al., 2007). Mn toxicity is particularly pronounced in acidic soils with a pH below 5.5. Under such conditions, the concentration of dissolved Mn^2+^ increases sharply, increasing Mn availability, which leads to phytotoxicity (Ducic and Polle, 2005). One such example is Guangxi, China, where soils are predominantly acidic and contain elevated levels of Mn, which limit plant growth and development (Chen, 2001). Sugarcane grown in the acidic soils of southern China often exhibits severe leaf chlorosis and stunted seedling growth owing to Mn toxicity (Long, 2020; Zhang et al., 2022).

Nitrogen (N) is essential for sugarcane growth and is required throughout its development cycle, as it plays a critical role in maintaining soil fertility and enhancing crop productivity (De Vries et al., 2022). The soil N cycle encompasses key biochemical processes, including mineralization, nitrification, denitrification, and biological nitrogen fixation (BNF), which collectively regulate nitrogen availability within ecosystems (Xiang, 2024). In soil, N exists in both organic and inorganic forms, with ammonium nitrogen (AN) and nitrate nitrogen (NN) being the primary forms available to plants (Robertson and Groffman, 2015). The transformation of soil N involves a series of microbially driven processes: mineralization converts organic N to ammonium, followed by nitrification and denitrification (Robertson and Groffman, 2015). Ammonia oxidation, which is the first step in nitrification, is mediated by specialized ammonia-oxidizing bacteria (AOB) and archaea (AOA) (Rütting et al., 2021). These microorganisms play a critical role in N use efficiency by regulating the conversion of ammonium to nitrate, the predominant form of N taken up by plants (Rütting et al., 2021). Notably, soil pH influences the dominance of nitrifying microbes; AOA tends to dominate in acidic soils, whereas AOB prevail in neutral to alkaline environments (Bai et al., 2022).

Current research has primarily examined the direct effects of Mn on plant photosynthesis, soil enzyme activity, N uptake, and plant metabolism (Wang et al., 2018; Gao et al., 2019; Yang et al., 2022). However, the link between Mn toxicity and microbially mediated N transformation, specifically the roles of ammonia-oxidizing bacteria (AOB) and archaea (AOA) in the regulation of nitrification, remains underexplored. This gap is significant because nitrification driven by AOB and AOA is the key process in converting ammonium (AN) to nitrate (NN), the primary form of N absorbed by sugarcane (Rütting et al., 2021). Notably, in acidic soils such as those in Guangxi, where AOA typically dominate and AOB are sensitive to pH fluctuations (Bai et al., 2022), Mn toxicity could disrupt these microbial communities, impair nitrification, and ultimately limit N availability. Furthermore, soil acidification, Mn toxicity, and flavonoid activity are closely interrelated. Flavonoids in the sugarcane rhizosphere may potentially inhibit Mn toxicity (Pang et al., 2025), while intercropping can reduce the accumulation of harmful elements in rhizosphere soil (Pang et al., 2022). Together, these reports further highlight the complex interactions between Mn toxicity, soil microbial processes, and nutrient cycling in this region.

Mechanistically, Mn toxicity may affect ammonia-oxidizing microorganisms through several pathways: redox interference, in which Mn²^+^ disrupts electron transport chains, inhibiting ammonia monooxygenase activity (Xu et al., 2017); pH-mediated stress, where Mn-induced acidification further suppresses pH-sensitive AOB (Nicol et al., 2008; Bai et al., 2015); and membrane damage, as excessive Mn increases microbial cell membrane permeability (Sieprawska et al., 2024). To address these knowledge gaps, this study aimed to answer two specific questions: (i) whether Mn toxicity suppresses soil nitrification by altering AOB/AOA abundance, activity, and structure and (ii) whether their differential sensitivity to Mn influences nitrate availability and sugarcane N uptake. We hypothesized that Mn toxicity in acidic soils reduces the nitrification potential by altering the abundance and community structure of AOB and AOA, with AOB being more sensitive to Mn due to their lower tolerance to acidic conditions and metal stress. This suppression of nitrification decreases NN availability, ultimately limiting sugarcane N uptake and utilization efficiency. Ultimately, this study sought to provide a targeted theoretical basis for optimizing N management strategies in sugarcane production systems under the constraints of acidic and Mn-rich soils.

## 2 Materials and methods

### 2.1 Experimental design

#### 2.1.1 Laboratory culture conditions under simulated soil Mn stress

The sugarcane variety Zhongzhe 9 was selected. Based on preliminary experiments with indoor culture and pot studies, the critical concentration for Mn toxicity symptoms in sugarcane was determined to be 328 mg·kg^-1^, with Mn supplied as anhydrous Mn sulfate (purity ≥ 99%). This concentration simulates extreme Mn accumulation in the acidic soils of southern China, where prolonged acidification and parent material weathering can lead to exchangeable Mn levels exceeding 300 mg·kg^-1^ in heavily affected sugarcane fields (Chen, 2001; Huang et al., 2016). For example, a soil survey reported over 40% of sugarcane fields in Guangxi had exchangeable Mn levels above 250 mg·kg^-1^, with heavily contaminated sites reaching 300–400 mg·kg^-1^ (Wu et al., 2015). The experiment included two N levels: N1 (0.14 g·kg^-1^) and N2 (0.28 g·kg^-1^), supplied as urea (N content 46%) and applied uniformly by dissolving it in deionized water and mixing it into the soil. Two Mn levels were established: −Mn (0 g·kg^-1^) and +Mn (328 mg·kg^-1^), resulting in five treatments: blank control CK (−N−Mn), T1 (N1−Mn), T2 (N1+Mn), T3 (N2−Mn), and T4 (N2+Mn). Treatments T1, T2, T3, and T4 received N; T1 and T3 excluded Mn toxicity, whereas T2 and T4 included it. Additionally, each treatment received 0.2 g of superphosphate (12% P_2_O_5_) and 0.3 g of potassium chloride (60% K_2_O) per kilogram of soil. All treatments were conducted in triplicate.

Acidic red soil used in the experiments was collected from the experimental base of the College of Agriculture, Guangxi University, China (22°51’N, 108°17’E). The basic physicochemical properties of the soil were as follows: total N (TN) 0.73 g·kg^-1^, total phosphorus (P) 0.87 g·kg^-1^, total potassium (K) 2.54 g·kg^-1^, AN 38.31 mg·kg^-1^, NN 20.95 mg·kg^-1^, alkaline hydrolysable N 74.06 mg·kg^-1^, available P 110.62 mg·kg^-1^, available K 134.78 mg·kg^-1^, exchangeable Mn^2+^ 6.83 mg·kg^-1^, easily reducible Mn^2+^ 27.05 mg·kg^-1^, soil organic matter (SOM) 9.87 g·kg^-1^, and pH 5.18. Soil texture determined using the hydrometer method revealed a clay loam texture composed of 42% sand, 28% silt, and 30% clay. The mineralogical composition analyzed by X-ray diffraction (XRD) indicated the presence of kaolinite, goethite, hematite, and quartz as the dominant minerals, which are typical of highly weathered acidic red soils in southern China. Soil from the 0–20-cm tillage layer was collected, air-dried, and passed through an 18-mesh sieve. After thorough mixing, 100.00 g of soil was weighed in a culture bottle, and the soil moisture was adjusted to 40% of the maximum field capacity. The culture bottles were sealed and 5–6 holes were punched into the cap to minimize soil moisture evaporation while ensuring an aerobic environment. All culture bottles were pre-incubated in the dark at 25°C for one week without shaking. Urea (N, 46%) was used as the N source, and Mn was added in the form of anhydrous Mn sulfate. Superphosphate (P_2_O_5_, 12%) was used as the P fertilizer and K chloride (K_2_O, 60%) as the K fertilizer. The fertilizers were added as described above, the soil moisture was adjusted to 60% of the maximum field capacity, and the incubation was continued. Deionized water was added daily to maintain a relatively constant moisture content.

#### 2.1.2 Pot experiment

The pot experiments were conducted in a glass greenhouse, following the same protocol as the laboratory culture experiments. Each pot was filled with 20.0 kg of air-dried soil that was passed through a 5 × 5-mm mesh sieve. N, P, and K fertilizers were mixed into the soil once according to the different treatments, and a Mn sulfate solution was added to the soil. Soil moisture was adjusted to 60% of the maximum field capacity and maintained for one week. Each pot contained two Zhongzhe 9 plants. Cuttings 2–3 cm in length were taken from single-node stems and soaked in water to encourage sprouting and rooting. Single-node stems with relatively consistent root and shoot growth were selected for transplantation. After 60 d of cultivation, sugarcane plants were harvested and analyzed to determine plant height, dry biomass, N uptake, and other related parameters. Each treatment was performed in triplicate.

Before harvesting, the height of the sugarcane plants was measured, and the entire plant was uprooted. After removing the larger soil clods, the rhizosphere soil attached to the root surface (approximately, 2 mm from the root surface) was gently brushed off into a sterile bag using a sterile brush. Brushes were sterilized by autoclaving at 121°C for 20 min before each use to prevent microbial contamination. The collected rhizosphere soil was divided into two portions: one was air-dried for measuring the basic physicochemical properties, while the other was stored at −80°C for microbial community structure and diversity analysis. Sugarcane plants were divided into roots and shoots, and each plant was stored separately in sterile bags. After washing with water and drying, samples were sterilized at 105°C, then dried in an oven at 60°C until constant weight was achieved. The dry matter content was measured and recorded, and the samples were crushed and sieved for N measurements.

### 2.2 Measurement items and methods

#### 2.2.1 Measurement of soil and sugarcane N, K, and P contents

The following were measured as previously described (Bao, 2000): soil pH (5:1 water-to-soil ratio) using the glass electrode method, soil available P using the 0.05 mol/L HCl–0.025 mol·L^-1^ 1/2 H_2_SO_4_ method, soil TN using the semi-micro Kjeldahl method, available K using the 1 mol·L^-1^ NH_4_OAc extraction–flame photometry method, alkaline hydrolyzable N using the alkaline hydrolysis diffusion method, soil AN using the 2 mol·L^-1^ KCl extraction–indophenol blue colorimetric method, soil exchangeable Mn using the 1 mol·L^-1^ NH_4_OAc extraction–atomic absorption spectrophotometry (AAS) method, and soil easily reducible Mn using the benzenediol–1 mol·L^-1^ NH_4_OAc extraction–AAS method. Soil NN was measured using dual-wavelength ultraviolet spectrophotometry, as previously described (Miao et al., 2019).

Sugarcane N content was measured as previously described (Bao, 2000). The plant samples were dried, crushed, and digested with H2SO4–H2O2. The N content was then measured using an automatic N analyzer, and the indicators were calculated as follows:


Sugarcane dry biomass=shoot dry biomass+root dry biomass

Sugarcane N uptake amount=N content×dry biomass

Sugarcane total N uptake amount=shoot N uptake amount+root N uptake amount

Sugarcane N uptake efficiency=[totalplant N accumulation/soil available N (fertilizer N+soil N)×100]

Sugarcane inorganic N content=AN content+NN content.

#### 2.2.2 Soil total DNA extraction, PCR amplification, and sequencing

Total DNA was extracted from soil samples using the conventional cetyltrimethylammonium bromide (CTAB) method. DNA integrity was determined using agarose gel electrophoresis, and DNA concentration was determined using a Nanodrop (Gao, 2014). An appropriate amount of DNA extract was placed in a centrifuge tube and diluted to 1 ng·μL^-1^ with sterile water. The diluted genomic DNA was then used as a template to synthesize specific barcoded primers according to the designated sequencing region, and PCR amplification was performed using the ABI GeneAmp^®^ 9700 PCR system (Thermo Fisher Scientific, Waltham, MA, USA). The PCR amplification reaction mixture was as follows: 4 μL of 5×FastPfu buffer, 2 μL of 2.5 mmol·L^-1^ dNTPs, 0.8 μL of 5 μmol·L^-1^ forward and reverse primers, 0.4 μL of Fast Pfu polymerase, 1 μL of 20 ng·μL^-1^ template DNA, and 0.2 μL of bovine serum albumin, and sterile water to reach a final volume of 20 μL. The PCR program was: S1, pre-denaturation at 95°C for 3 min, one cycle; S2, denaturation at 95°C for 30 s, annealing at 55°C for 30 s, extension at 72°C for 30 s, 27 cycles; S3, extension at 72°C for 10 min, one cycle.

Real-time fluorescence quantitativem PCR (qPCR) analysis of *AOB-amoA* and *AOA-amoA* genes was performed using the following primers and sequences:

*AOB-amoA*: baom1F (5′-GGGGTTTCTACTGGTGGT-3′), bamoA2R (5′-CCCCTCKGSA AAGCCTTCTTC-3′).
*AOA-amoA*: amoAF (5′-STAATGGTCTGGCTTAGACG-3′), amoAR (5′GCGGCCATCCATCTGTATGT-3′).

The reaction system was 20 μL, including 10 μL TransStart TipTOP Green qPCR SuperMix, 1 μL DNA template (50 ng·μL^-1^), 0.8 μL upstream and downstream primers (10 μmol·L^-1^), 2 μL bovine serum protein (20 mg·mL^-1^), 0.4 μL ROXDYE II and 5 μL sterile water. The PCR procedure was pre-denaturation at 94°C for 5 min (to fully unwind DNA strands), followed by 45 cycles of denaturation at 94°C for 30 s (separate double-stranded DNA), annealing at 53°C for 30 s (allow primers to bind to templates), and extension at 72°C for 30 s (synthesize new DNA strands). Final hold at 4°C.

The PCR products were analyzed by agarose gel electrophoresis. Equal amounts of the samples were mixed according to the concentrations of the PCR products. Fully mixed PCR products were purified using 1×TAE 2% agarose gel electrophoresis, excised, and recovered using an Axyprep DNA Gel Recovery Kit (AXYGEN, Union City, CA, USA). After purification, the samples were sent to Majorbio Bio-Pharm Technology (Shanghai, China) for high-throughput sequencing using a MiSeq PE300 platform (Illumina, San Diego, CA, USA).

#### 2.2.3 Bioinformatics analysis and data processing

Raw sequencing data were subjected to quality control using established protocols via the following steps: (1) trimming of bases with quality scores< 20 at read termini; (2) removal of reads shorter than 50 bp after trimming and those containing ambiguous N bases; (3) assembly of paired-end reads based on overlap relationships using FLASH software (Magoč and Salzberg, 2011) with a minimum overlap length of 10 bp and a maximum allowed mismatch ratio of 0.2 in overlapping regions, with non-compliant sequences discarded; (4) sample demultiplexing according to barcode and primer sequences, with subsequent adjustment of sequence orientation. Barcodes required exact matches, whereas up to two mismatches were permitted in the primer sequences.

High-quality sequences were clustered into operational taxonomic units (OTUs) at a 97% similarity threshold using the UPARSE algorithm (Edgar, 2013), which simultaneously removes singleton reads and chimeric sequences during clustering. Taxonomic assignment of each OTU was performed using the RDP Classifier (Wang et al., 2007) with a confidence threshold of 0.8 to obtain species-level classification information.

### 2.3 Statistical analysis

Data analysis and graph plotting were performed using Microsoft Excel 2016. Three biological replicates and three technical repeats were performed for each treatment. Treatment differences were analyzed by one-way analysis of variance (ANOVA) followed by Duncan’s new multiple-range test at *P* < 0.05, using IBM SPSS Statistics 24 (IBM Corp., Armonk, NY, USA). Redundancy analysis (RDA) was performed with Monte Carlo permutation tests to determine the significance of environmental factors, using Canoco 5.0 software. Analysis and mapping of Microbial communities were analyzed and mapped using the Majorbio Cloud platform (https://www.majorbio.com/).

## 3 Results

### 3.1 Effect of Mn toxicity on the physicochemical properties and ammonia-oxidizing microbial communities of sugarcane rhizosphere soil

#### 3.1.1 Physicochemical properties

**Figure 1:**
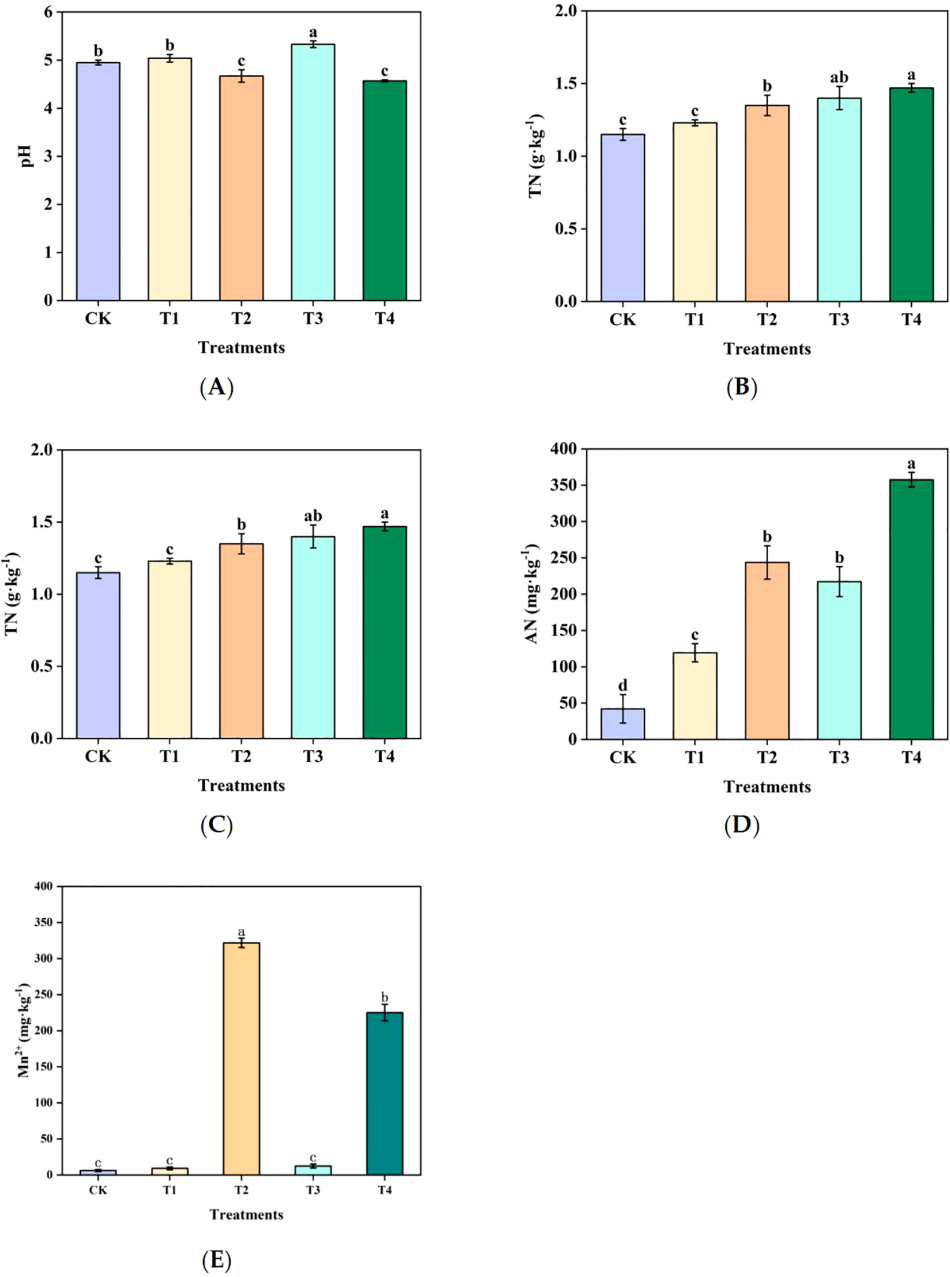
Effects of Mn on the physicochemical properties of sugarcane rhizosphere soil: **(A)** pH, **(B)** TN, **(C)** NN, **(D)** AN, **(E)** Mn^2+^. Different letters indicate significant differences (*P* < 0.05).

Compared with CK, soil pH value decreased by 0.2–0.3 units in T2 and T4, whereas it increased slightly in T1 and T3 (**Figure 1A**). Soil TN content increased across all treatments, with significant increases observed in T3 and T4 (**Figure 1B**). Soil AN levels were significantly elevated in all treatments, with T2 and T4 exhibiting higher AN concentrations than those of T1 and T3 (**Figure 1D**). In contrast, soil NN levels were suppressed under Mn toxicity, as T2 and T4 exhibited lower NN levels than those of T1 and T3 (**Figure 1C**). Additionally, the exchangeable Mn levels were significantly higher in T2 and T4 than in T1 and T3, indicating that Mn toxicity enhanced Mn availability in the soil (**Figure 1E**).

#### 3.1.2 Ammonia-oxidizing microorganisms

##### 3.1.2.1 Soil nitrification potential and the abundance of ammonia-oxidizing microorganisms

**Figure 2:**
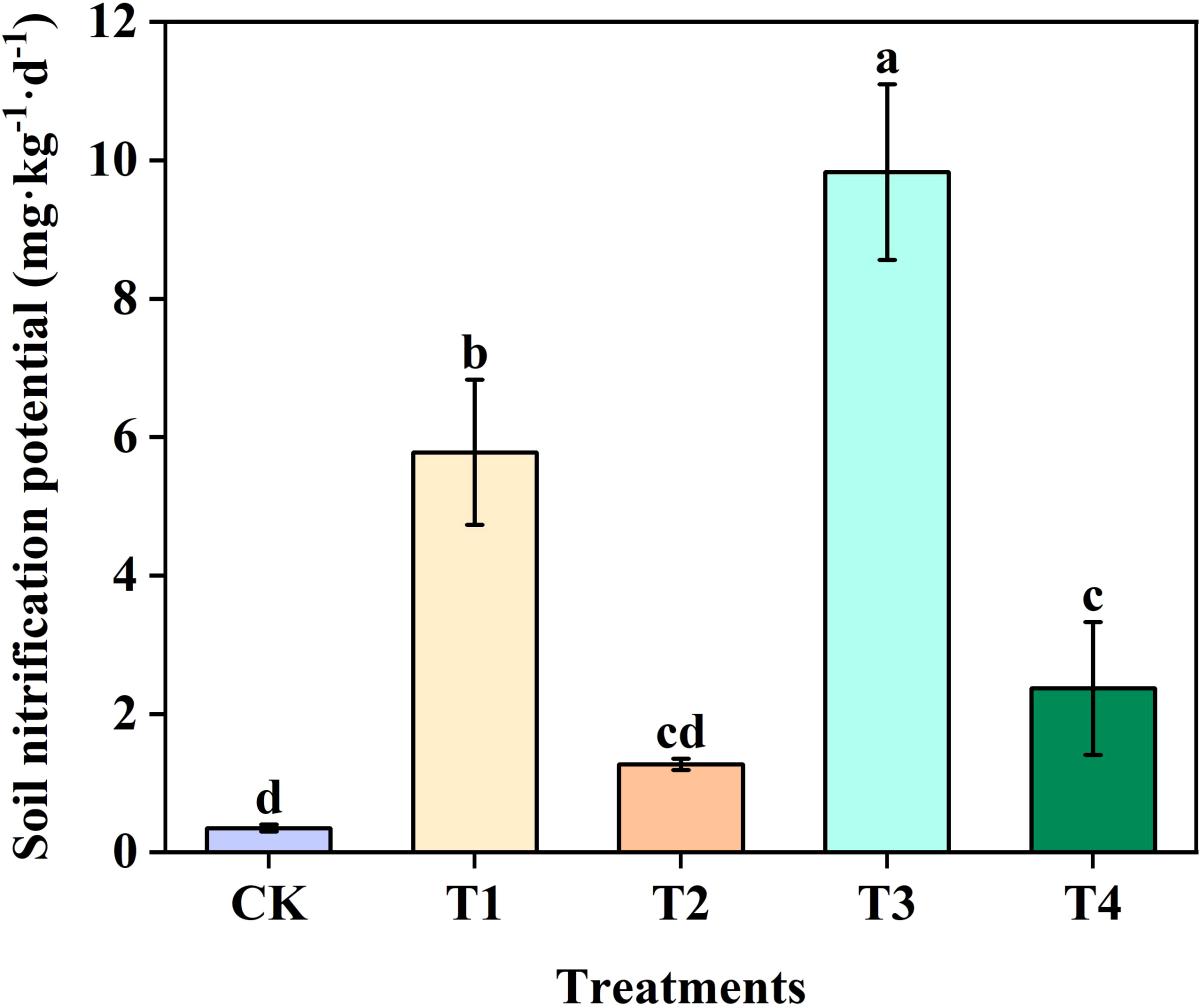
Effect of different treatments on nitrification potential of sugarcane rhizosphere soil. Different letters indicate significant differences (*P* < 0.05).

Soil nitrification potential across all treatments ranged from 0.35 to 9.83 mg·kg^-1^d^-1^. At the same N level, nitrification potential in T2 was significantly lower than that in T1, and that in T4 was significantly lower than that in T3. Specifically, nitrification potential in T2 was 78.03% lower than that in T1, indicating a pronounced suppression of nitrification (**Figure 2**).

The abundance of AOB amoA genes in sugarcane rhizosphere soil ranged from 2.16×10^5^ to 5.75×10^5^ copies per gram of dry soil, while AOA amoA genes abundance ranged from 0.31×10^6^ to 2.98×10^6^ copies per gram of dry soil (**Table 1**). Thus, the copy numbers of AOA amoA were higher than those of AOB amoA. At the same N level, AOB amoA genes abundance in T2 and T4 was significantly lower than that in T1 and T3, with reductions of 44.87% in T2 compared to T1, and 46.50% in T4 compared to T3. This substantial decrease indicates that Mn toxicity strongly inhibits AOB growth and activity. In contrast, the abundance of AOA amoA genes did not differ significantly between the two N levels, regardless of Mn treatment. The AOA/AOB ratio ranged from 1.15 to 13.78 across all treatments, with no significant differences between N levels or Mn treatments. Overall, these findings suggest that Mn toxicity suppresses nitrification capacity primarily by suppressing the abundance and activity of ammonia-oxidizing microorganisms, thereby impeding nitrification in acidic soils.

**Table 1:** Influence of different treatments on the abundance and ratio of AOB and AOA.

Treatments	*AOB-amoA* gene copy number (10^5^)/g dry soil	*AOA-amoA* gene copy number (10^6^)/g dry soil	AOA/AOB
CK	2.16 ± 0.29^d^	2.98 ± 0.51^a^	13.78 ± 0.98^a^
T1	5.75 ± 0.78^a^	1.09 ± 0.13^b^	1.92 ± 0.46^bc^
T2	3.17 ± 0.46^c^	0.88 ± 0.18^b^	2.83 ± 0.82^b^
T3	4.28 ± 0.44^b^	0.48 ± 0.07^c^	1.15 ± 0.26^c^
T4	2.29 ± 0.47^cd^	0.31 ± 0.05^c^	1.41 ± 0.56^c^

Data are expressed as the mean ± standard deviation. Different letters indicate significant differences (*P<* 0.05).

##### 3.1.2.2 Ammonia-oxidizing microorganisms diversity

At the same nitrogen level, the Shannon diversity index of AOB in T2 showed no significant difference compared to that in T1 at the N1 level, whereas the Shannon diversity index of AOB in the T4 treatment was significantly higher than that in the T3 treatment at the N2 level (*P* < 0.05). The Simpson index of AOB in the T1 and T2 treatments at the N1 level was not significantly different, whereas the Simpson index of AOB in the T4 treatment at the N2 level was significantly lower than that in the T3 treatment (*P* < 0.05). Moreover, no significant differences in AOB Chao1 or ACE indices were observed between treatments with or without Mn at either N level. In contrast, the Shannon, Simpson, Chao1, and ACE indices of AOA did not show significant differences between the two N levels, regardless of Mn toxicity (**Table 2**). These findings suggest that Mn toxicity may moderately enhance the diversity of the AOB community, particularly under high-N conditions, while exerting minimal influence on the diversity of the AOA community.

**Table 2:** Effect of Mn on the operational taxonomic unit (OTU) number and alpha diversity indices of soil AOB and AOA communities.

AOB/AOA	Treatments	Shannon	Simpson	ACE	Chao1	Coverage
AOB	CK	1.89 ± 0.10^a^	0.30 ± 0.04^c^	25.00 ± 1.00^a^	24.67 ± 0.58^a^	1.000 ± 0.000^a^
	T1	0.96 ± 0.19^b^	0.65 ± 0.07^b^	28.88 ± 10.26^a^	28.50 ± 9.58^a^	1.000 ± 0.000^b^
	T2	0.91 ± 0.08^b^	0.66 ± 0.05^b^	27.42 ± 5.25^a^	27.06 ± 5.08^a^	1.000 ± 0.000^ab^
	T3	0.63 ± 0.02^c^	0.77 ± 0.01^a^	23.21 ± 3.92^a^	22.78 ± 3.67^a^	1.000 ± 0.000^ab^
	T4	0.89 ± 0.10^b^	0.65 ± 0.05^b^	20.65 ± 1.97^a^	20.00 ± 3.00^a^	1.000 ± 0.000^ab^
AOA	CK	2.87 ± 2.87^a^	0.10 ± 0.03^a^	73.72 ± 4.76^a^	73.75 ± 4.68^a^	0.999 ± 0.000^a^
	T1	2.96 ± 2.96^a^	0.09 ± 0.02^a^	74.55 ± 2.65^a^	74.53 ± 3.50^a^	0.999 ± 0.000^a^
	T2	2.84 ± 2.84^a^	0.10 ± 0.00^a^	71.79 ± 3.59^a^	72.08 ± 1.84^a^	1.000 ± 0.000^a^
	T3	2.89 ± 2.89^a^	0.10 ± 0.02^a^	70.93 ± 1.83^a^	70.65 ± 1.46^a^	1.000 ± 0.000^a^
	T4	2.82 ± 2.82^a^	0.11 ± 0.03^a^	73.66 ± 3.43^a^	74.38 ± 2.07^a^	0.999 ± 0.000^a^

Data are expressed as the mean ± standard deviation. Different letters indicate significant differences (*P* < 0.05).

##### 3.1.2.3 Principal component analysis of ammonia-oxidizing microorganisms communities under different Mn treatments

**Figure 3:**
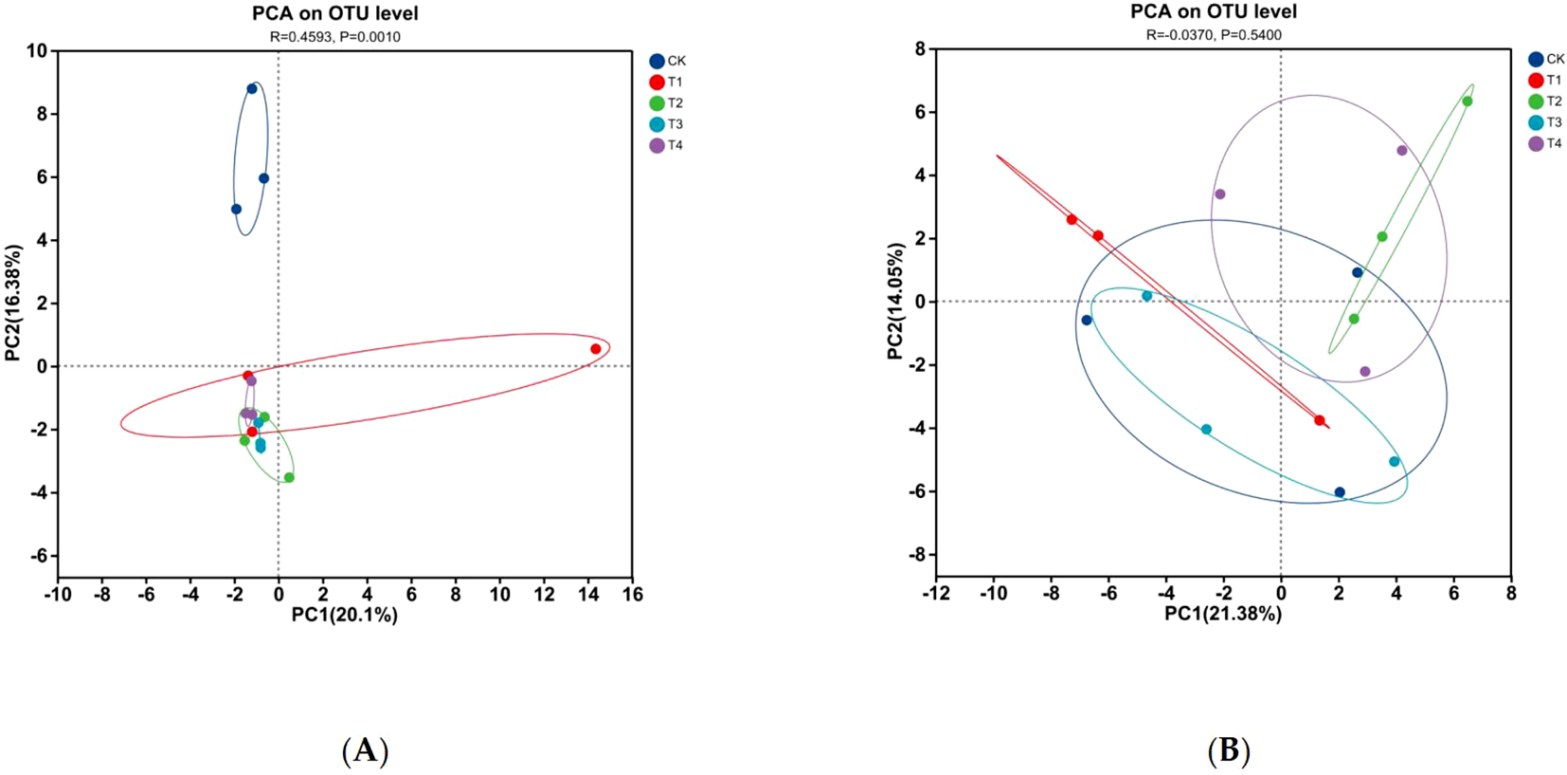
Principal component analysis (PCA) of AOB and AOA community composition: investigating the differences among different treatments: **(A)** AOB, **(B)** AOA.

The PCA revealed that both AOB and AOA communities exhibited clustering patterns primarily determined by N levels, with Mn treatments exerting limited influence on the overall community structure (**Figure 3**). This N-driven segregation accounted for the majority of the observed variance, as confirmed by PERMANOVA, which indicated that the N level explained 62.1% of the total variance in AOB communities (*P* = 0.001). Within each N level, the Mn-treated and corresponding control samples demonstrated considerable overlap in the PCA ordination space, indicating that Mn toxicity alone did not substantially alter the overall composition of AOB communities.

Notably, further statistical evaluation using PERMANOVA revealed nuanced effects of Mn toxicity on community composition (**Table 3**). Although the main effect of Mn toxicity on AOB was not statistically significant (*R²* = 0.070, *P* = 0.089), the interaction between N level and Mn toxicity explained 5.0% of the variance (*P* = 0.132), suggesting that the effect of Mn may be modulated by nitrogen availability. The spatial configuration in the PCA plots, characterized by the close clustering between the Mn-treated and control samples within each N level (**Figure 3A**), further indicates that the N regime exerts a predominant influence on AOB community assembly, overriding the effects of Mn toxicity.

**Table 3:** Permutational multivariate analysis of variance (PERMANOVA) of AOB and AOA community composition.

AOB/AOA	Factor	Df	SumsOfSqs	MeanSqs	F_Model	*R^2^*	*P*
AOB	N level	1	0.1892	0.1892	28.76	0.621	0.001
	Mn Toxicity	1	0.0215	0.0215	3.27	0.070	0.089
	N level × Mn Toxicity	1	0.0153	0.0153	2.33	0.050	0.132
	Residuals	11	0.0724	0.0066	–	0.259	–
	Total	14	0.30425	–	–	1.000	–
AOA	N level	1	0.1085	0.1085	4.73	0.302	0.045
	Mn Toxicity	1	0.0423	0.0423	1.84	0.118	0.191
	N level × Mn Toxicity	1	0.0291	0.0291	1.26	0.081	0.283
	Residuals	11	0.2519	0.0229	–	0.699	–
	Total	14	0.3598	–	–	1.000	–

In contrast, AOA communities demonstrated even greater structural stability under the experimental conditions. PERMANOVA results indicated that neither N level (*R²* = 0.302, *P* = 0.045) nor Mn toxicity (*R²* = 0.118, *P* = 0.191) substantially influenced AOA community composition. The extensive overlap among all treatments in the PCA ordination (**Figure 3B**), coupled with a non-significant interaction term (*R²* = 0.081, *P* = 0.283), supports the interpretation that AOA communities maintained their structural integrity despite variations in both N and Mn levels. Overall, these results demonstrated that N availability is the primary determinant of community differentiation among ammonia-oxidizing microorganisms, whereas Mn toxicity induces only subtle compositional shifts, with AOB exhibiting greater sensitivity to these environmental stressors than AOA.

##### 3.1.2.4 Ammonia-oxidizing microorganisms community composition

**Figure 4:**
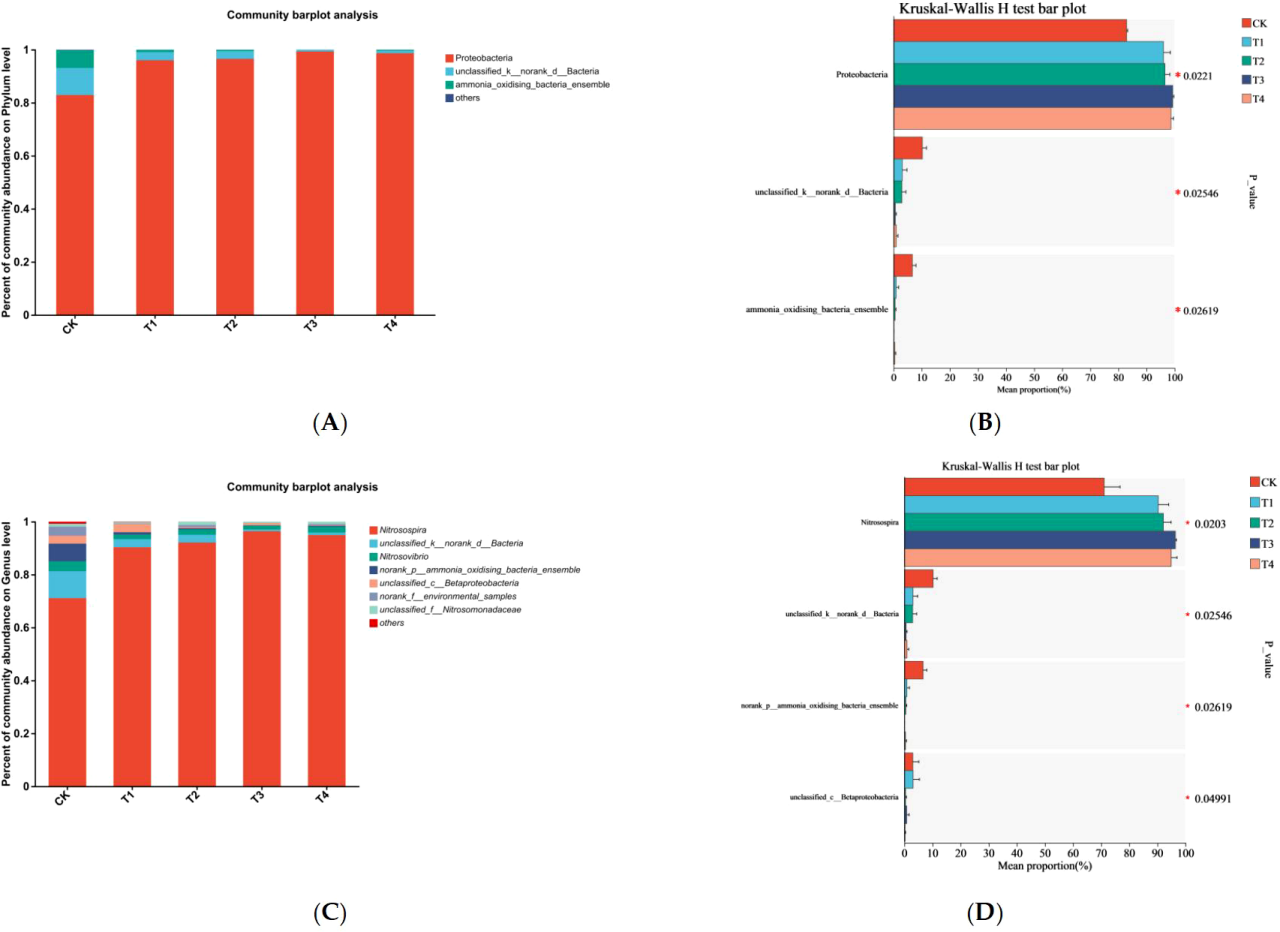
Ammonia-oxidizing bacteria (AOB) community composition under different treatments: **(A)** AOB community composition at the phylum level in soil, **(B)** Differences in AOB taxa at the phylum level in soil, **(C)** AOB community composition at the genus level in soil, **(D)** Differences in AOB taxa at the genus level in soil. * means significant at *P* ≤ 0.05. ** means significant at *P* ≤ 0.01. *** means significant at *P* ≤ 0.001.

At the phylum level, the AOB community was predominantly composed of Proteobacteria, with relative abundances ranging from 82.9% to 99.3%. Additionally, *Unclassified_k_norank_d_Bacteria* accounted for 0.06% to 10.21%, while the *ammonia_oxidising_Bacteria_ensemble* represented 0.09% to 6.65%, suggesting additional contributions to nitrification, although their lower abundance indicates a secondary role compared to Proteobacteria. At the same N level, there were no significant differences observed regarding the relative abundance of AOB at the phylum level between the T2 and T1 treatments, or between the T4 and T3 treatments (**Figures 4A, B**, **Table 4**).

**Table 4:** The top 10 phylum level AOB of soil relative abundance under different treatments.

Species name	CK (%)	T1 (%)	T2 (%)	T3 (%)	T4 (%)
Phylum level					
Proteobacteria	82.9 ± 0.4^b^	96.0 ± 2.5^a^	96.5 ± 1.7^a^	99.3 ± 0.4^a^	98.7 ± 0.9^a^
unclassified_k_norank_d_Bacteria	10.2 ± 1.5^a^	3.1 ± 1.7^b^	2.9 ± 1.4^b^	0.6 ± 0.4^c^	0.9 ± 0.6^c^
ammonia_oxidising_Bacteria_ensemble	6.7 ± 1.3^a^	0.9 ± 0.9^b^	0.5 ± 0.3^b^	0.1 ± 0.0^c^	0.4 ± 0.3^bc^
Genus level					
*Nitrosospira*	71.1 ± 5.6^c^	90.3 ± 3.7^ab^	92.1 ± 2.8^a^	96.4 ± 0.4^a^	94.9 ± 2.1^a^
*unclassified_k_norank_d_Bacteria*	10.2 ± 1.5^a^	3.1 ± 1.7^b^	1.2 ± 1.4^c^	0.6 ± 0.4^c^	0.9 ± 0.6^c^
*Nitrosovibrio*	3.7 ± 3.0^a^	1.7 ± 0.7^a^	2.0 ± 0.4^a^	1.5 ± 0.3^a^	2.3 ± 1.9^a^
*norank_p_ammonia_oxidising_bacteria_ensemble*	6.7 ± 1.3^a^	0.9 ± 0.9^b^	0.5 ± 0.3^b^	0.1 ± 0.0^c^	0.4 ± 0.3^bc^
*unclassified_c_Betaproteobacteria*	3.0 ± 2.1^a^	3.1 ± 2.3^a^	0.4 ± 0.3^b^	0.8 ± 0.7^b^	0.2 ± 0.2^b^
*norank_f_environmental_samples*	3.4 ± 4.6^a^	0.2 ± 0.2^b^	0.8 ± 0.0^b^	0.1 ± 0.1^b^	0.4 ± 0.4^b^
*unclassified_f_Nitrosomonadaceae*	1.1 ± 0.5^a^	0.5 ± 0.3^b^	1.2 ± 1.0^a^	0.4 ± 0.3^b^	0.9 ± 0.6^ab^

Data are expressed as the mean ± standard deviation. Different letters indicate significant differences (*P* < 0.05).

At the genus level, the dominant AOB genus was *Nitrosospira* (71.1%–96.4%). Other low-abundance genera (< 3%) that were not significantly affected by Mn toxicity are not discussed further. Under the same nitrogen levels, except for *unclassified_c_Betaproteobacteria* at the N1 level, the relative abundance was significantly lower in T2 than in T1, and no significant differences were observed in the other treatments (**Figures 4C, D**, **Table 5**).

**Table 5:** The top 10 phylum level AOA of soil relative abundance under different treatments.

Species name	CK (%)	T1 (%)	T2 (%)	T3 (%)	T4 (%)
Phylum level					
Crenarchaeota	40.9 ± 4.0^a^	39.6 ± 5.0^a^	33.6 ± 6.3^b^	42.3 ± 5.0^a^	32.4 ± 4.0^b^
environmental_samples_k:norank_d:Archaea	29.6 ± 8.7^b^	33.2 ± 4.6^b^	43.5 ± 5.1^a^	28.2 ± 7.4^b^	37.6 ± 10.3^ab^
unclassified_k:norank_d:Archaea	24.9 ± 8.0^a^	23.9 ± 3.0^a^	19.0 ± 0.9^b^	27.2 ± 5.3^a^	26.9 ± 8.7^a^
Thaumarchaeota	4.6 ± 0.7^a^	3.3 ± 0.6^b^	3.9 ± 0.6^ab^	2.4 ± 0.3^c^	3.1 ± 0.3^bc^
Genus level					
*norank_c:environmental_samples_p:Crenarchaeota*	40.9 ± 4.0^a^	39.6 ± 5.0^a^	33.6 ± 6.3^b^	42.3 ± 5.0^a^	32.4 ± 4.0^b^
*norank_p:environmental_samples_k:norank*	29.6 ± 8.7^b^	33.2 ± 4.6^b^	43.5 ± 5.1^a^	28.2 ± 7.4^b^	37.6 ± 10.3^ab^
*unclassified_k:norank_d:Archaea*	24.9 ± 8.0^a^	23.9 ± 3.0^a^	19.0 ± 0.9^b^	27.2 ± 5.3^a^	26.9 ± 8.7^a^
*Nitrososphaera*	4.6 ± 0.7^a^	3.3 ± 0.6^b^	3.9 ± 0.6^ab^	2.4 ± 0.3^c^	3.1 ± 0.3^bc^

Data are expressed as the mean ± standard deviation. Different letters indicate significant differences (*P* < 0.05).

**Figure 5:**
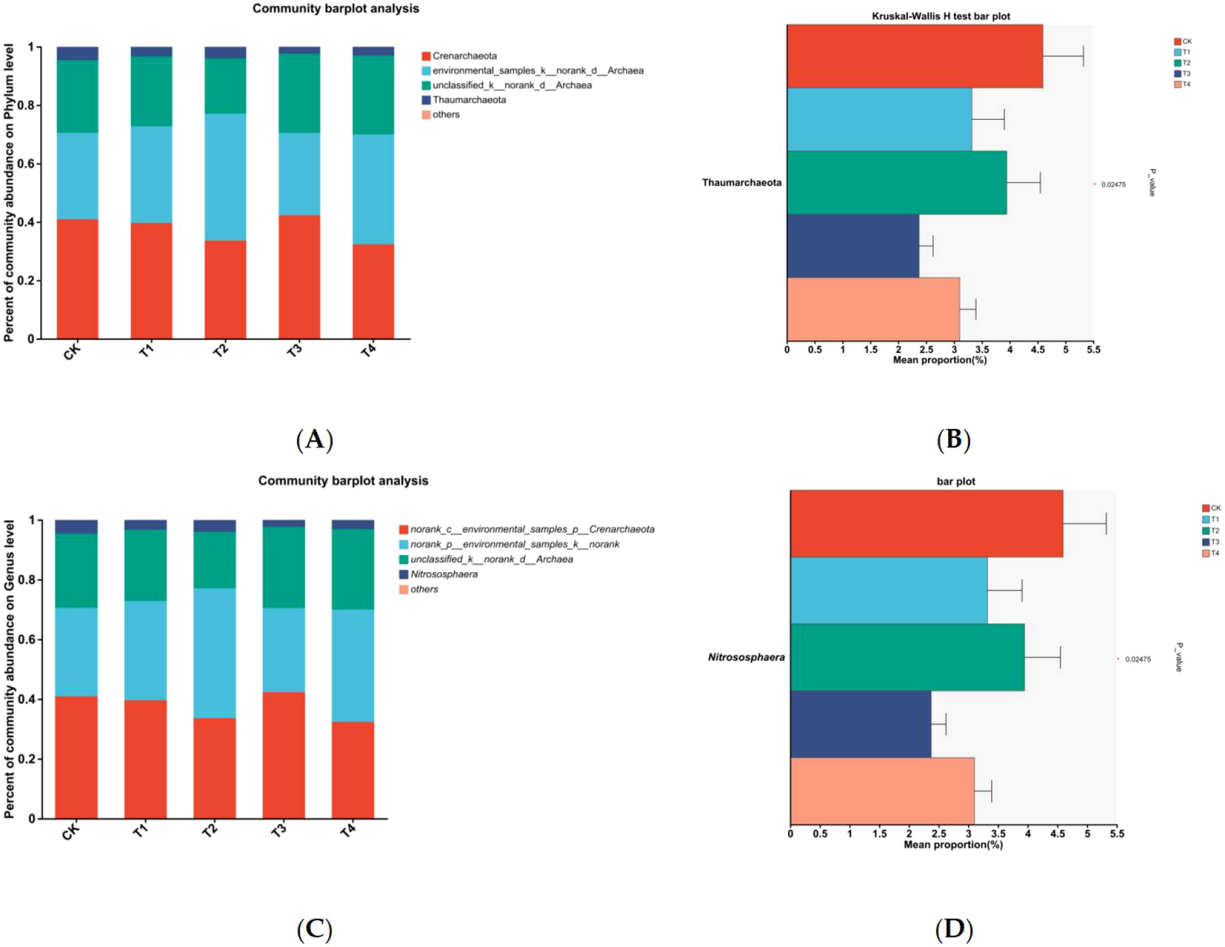
Ammonia-oxidizing archaea (AOA) community composition under different treatments: **(A)** AOA community composition at the phylum level in soil, **(B)** Differences in AOA taxa at the phylum level in soil, **(C)** AOA community composition at the genus level in soil, **(D)** Differences in AOA taxa at the genus level in soil. * means significant at *P* ≤ 0.05. ** means significant at *P* ≤ 0.01. *** means significant at *P* ≤ 0.001.

At the phylum level, the AOA community was dominated by the phyla *Crenarchaeota*, *environmental_samples_k_norank_d_Archaea*, and *unclassified_k_norank_d_Archaea*, with relative abundances ranging from 32.3% to 42.3%, 28.2% to 43.4%, and 19.0% to 27.2%, respectively (**Figure 5A**, **Table 4**). Under the same nitrogen level, except for a significant increase in *Thaumarchaeota* in the T4 treatment compared to that in the T3 treatment at the N2 level, no significant differences were observed in the other treatments (**Figure 5B**).

At the genus level, the dominant AOA genera were *norank_c_environmental_samples_p_Crenarchaeota*, *norank_p_environmental_samples_k_norank*, *unclassified_k_norank_d_Archaea*, and *Nitrososphaera*, with relative abundances ranging from 32.3%–42.3%, 28.2%–43.5%, 19.0%–7.2%, and 2.4%–4.6%, respectively. Under the same N level, except for a significant increase in Nitrososphaera in the T4 treatment compared with the T3 treatment at the N2 level, no significant differences were observed across the treatments (**Figures 5C, D**). In summary, Mn toxicity had a relatively minor effect on the composition and structure of soil AOB and AOA communities.

### 3.2 Effects of Mn toxicity on sugarcane growth, development, and N uptake, and N utilization

#### 3.2.1 Sugarcane growth and development

**Figure 6:**
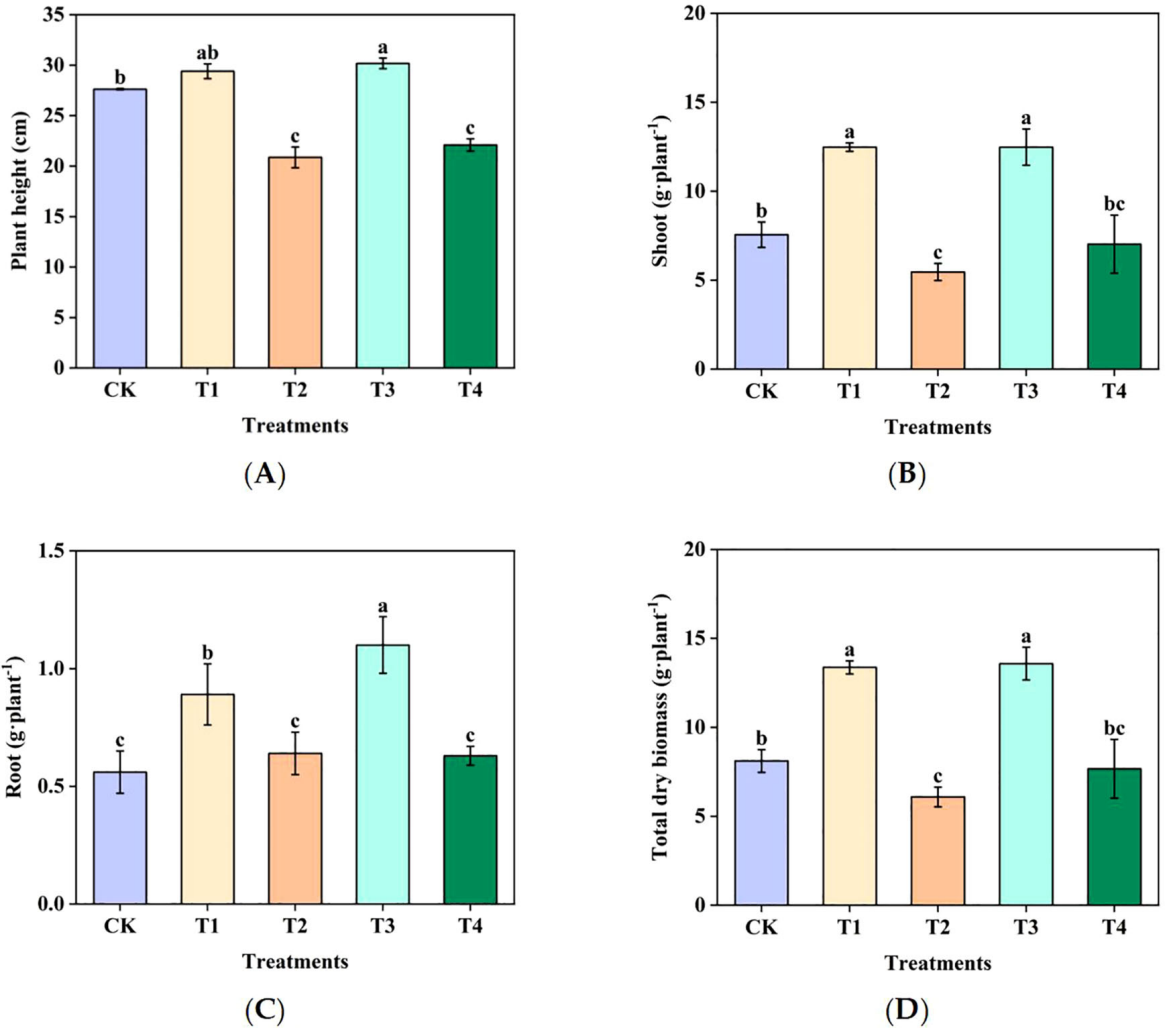
Effects of manganese on the plant height and dry biomass of sugarcane: a comparison among different treatments: **(A)** Plant height, **(B)** dry biomass (shoot), **(C)** dry biomass (root), and **(D)** dry biomass (total dry biomass). Different letters indicate significant differences (*P* < 0.05).

Compared to CK, T2 and T4 exhibited significant decreases in sugarcane plant height and dry biomass, whereas T1 and T3 showed significant increases in sugarcane plant height and dry biomass (**Figure 6**). Among them, the T2 treatment decreased by 54.5% compared to the T1 treatment (aboveground treatment decreased by 56.3%, root system decreased by 28.1%), and the T4 treatment decreased by 43.5% compared to the T3 treatment (aboveground treatment decreased by 43.7% and root system decreased by 42.7%). These findings indicate that Mn toxicity significantly reduces dry biomass and inhibits sugarcane growth.

#### 3.2.2 Sugarcane N uptake and utilization

**Figure 7:**
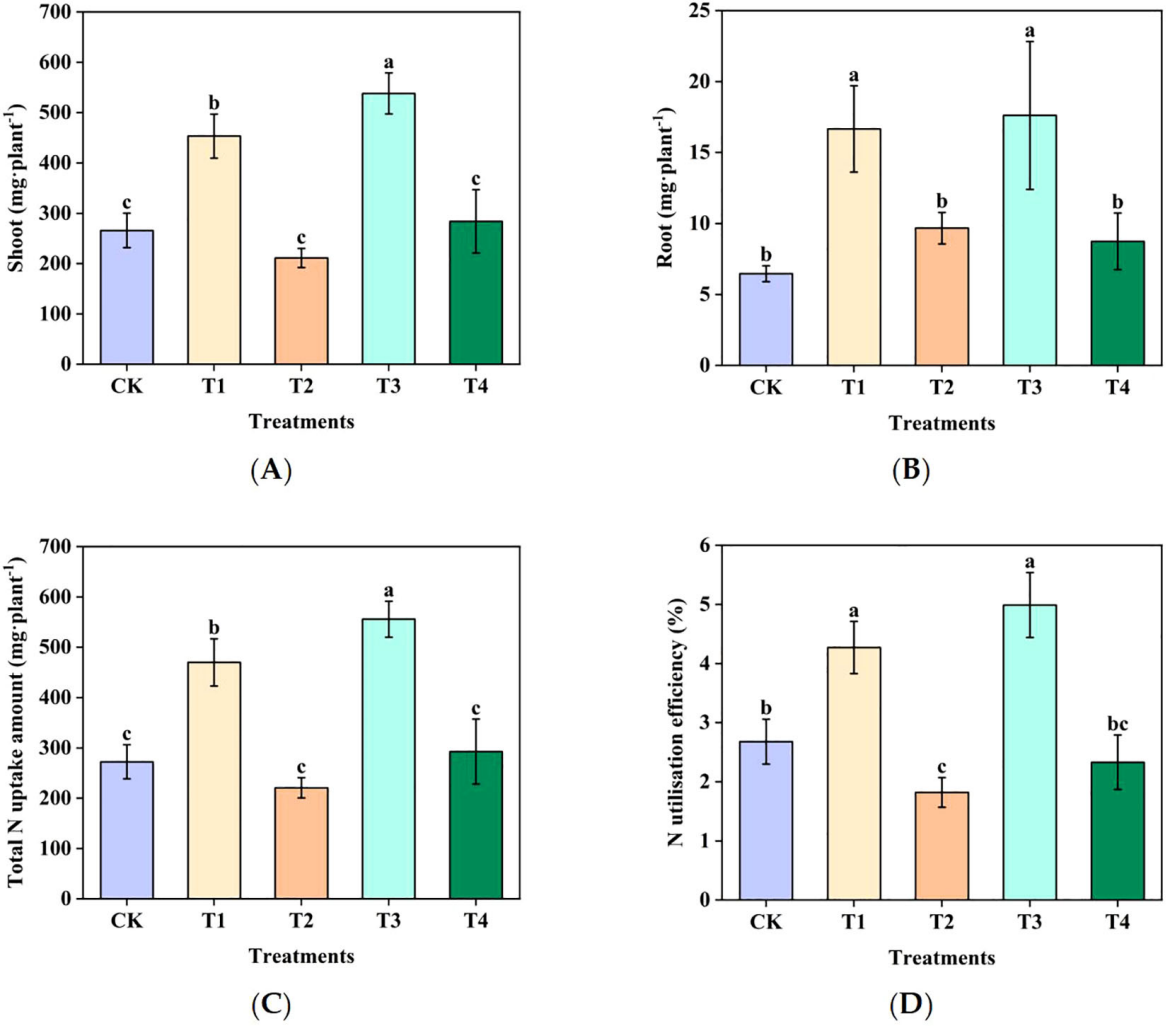
Effect of Mn toxicity on sugarcane nitrogen uptake and utilization in sugarcane: analysis of the differences in nitrogen uptake amounts and utilization efficiencies under various treatments: **(A)** N uptake amount, **(B)** N uptake amount (root), **(C)** N uptake amount (total N uptake amount), and **(D)** N utilization efficiency. Different letters indicate significant differences (*P* < 0.05).

Treatments T2 and T4 showed significantly lower N uptake than T1 and T3; shoot, root, and total N uptake amounts and efficiency were 53.4%, 42.0%, 53.0%, and 57.4% lower in T2 than in T1, respectively, and 47.3%, 50.4%, 47.4%, and 53.3% lower in T4 than in T3, respectively (**Figure 7**). These findings indicate that Mn toxicity significantly inhibited N uptake and accumulation in sugarcane, ultimately reducing both N uptake and utilization efficiency.

### 3.3 Correlation analysis of soil physicochemical properties and sugarcane N utilization efficiency with the ammonia-oxidizing microorganisms community structure and diversity

Correlation analysis results revealed that the soil nitrification potential was significantly and positively correlated with soil pH, NN content, N accumulation, and uptake efficiency in sugarcane roots, shoots, and the entire plant (**Table 6**). Conversely, nitrification potential was significantly negatively correlated with soil organic carbon content. The AOB amoA gene abundance exhibited significant positive correlations with soil pH and NN content, as well as with N accumulation and uptake efficiency in sugarcane. In contrast, AOA amoA gene abundance was significantly and positively correlated with soil organic carbon content, but showed significant negative correlations with TN and AN. Additionally, the AOA/AOB ratio was positively correlated with N accumulation in sugarcane roots and soil organic carbon content, but negatively correlated with TN and AN.

**Table 6:** Correlation analysis of soil nitrification potential and amoA gene abundance in AOB and AOA communities with soil physical and chemical factors.

AOB/AOA	Analysis Index	pH	SOC	TN	NO_3_^–^N	NH_4_^+^-N	Mn^2+^	NUS	NUR	TNU	NUE
	nitrification potential	0.774**	-0.679**	0.141	0.958**	0.077	-0.478	0.915**	0.71**	0.961**	0.836**
AOB	amoB gene	0.578*	-0.366	-0.113	0.599*	-0.202	-0.396	0.665**	0.666**	0.670**	0.702**
AOA	amoA gene	0.111	0.797**	-0.791**	-0.392	-0.836**	-0.419	-0.305	-0.423	-0.312	-0.130
	AOA/AOB	0.009	0.767**	-0.709**	-0.454	-0.721**	-0.334	-0.400	-0.540*	-0.408	-0.248

* means significant at *P* ≤ 0.05, ** means significant at *P* ≤ 0.01.

**Figure 8:**
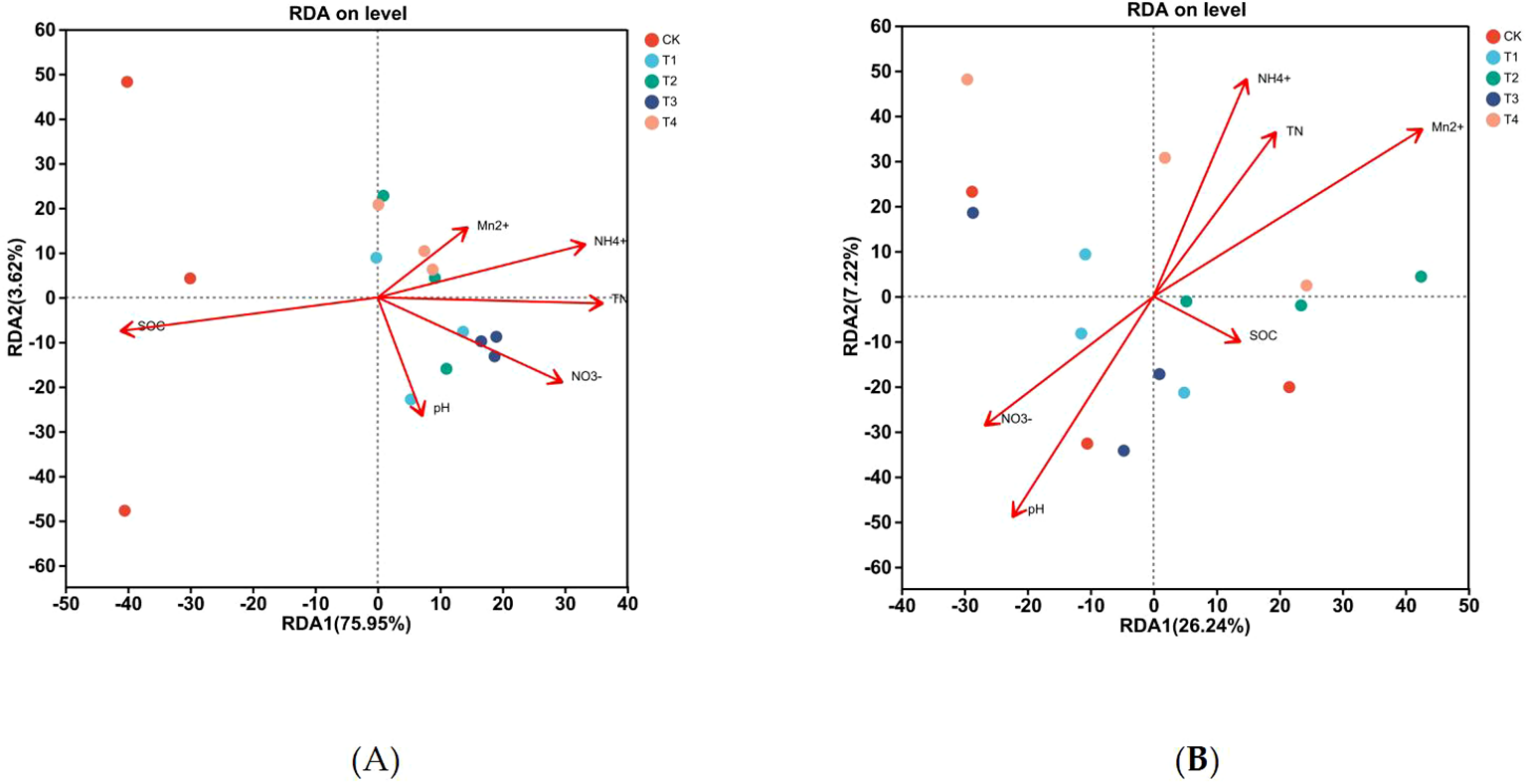
Redundancy analysis (RDA) of soil AOB and AOA communities and physicochemical properties: Revealing the Interrelationships under Mn Toxicity: **(A)** AOB; **(B)** AOA.

Redundancy analysis (RDA) was conducted on soil physicochemical factors and AOA and AOB communities at the OTU level (**Figure 8**). Axis 1 and 2 collectively accounted for 79.58% and 33.46% of the variation in AOB and AOA communities, respectively. Soil organic carbon (SOC) and TN were the most prominent driving factors in the AOB community. The order of their impact strength was determined as: soil organic carbon > TN > AN > NN > pH > Mn²^+^. Specifically, soil organic carbon (*R²* = 0.6928, *P* = 0.002) and total nitrogen (*R²* = 0.4876, *P* = 0.008) exerted highly significant effects on the AOB community structure, whereas AN (*R²* = 0.5005, *P* = 0.012) also showed a significant impact. This highlights the fact that the AOB community is more sensitive to changes in soil carbon and N nutrient status, reflecting an indirect regulatory pathway through which environmental factors shape AOB communities.

In contrast, the AOA community was primarily influenced by exchangeable Mn²^+^, followed by pH and AN, with the impact order being: Mn²^+^ > pH > AN > TN > NN > SOC. Among these, exchangeable Mn²^+^ (*R²* = 0.6469, *P* = 0.006) had a highly significant effect on the AOA community, and both pH (*R²* = 0.5144, *P* = 0.018) and AN (*R²* = 0.4191, *P* = 0.037) also exerted significant impacts. This indicated that AOA communities were more directly affected by soil Mn toxicity and acidification, which differed from the regulatory patterns observed for AOB. This finding indicates that Mn toxicity inhibits the activity of ammonia-oxidizing microorganisms, weakens soil nitrification, affects soil N transformation, and ultimately reduces N uptake and utilization in sugarcane.

## 4 Discussion

### 4.1 Effects of Mn toxicity on the physicochemical properties and microbial communities of rhizosphere soil

This study provides novel insights into the mechanisms by which Mn toxicity disrupts soil N cycling, particularly through differential effects on AOB and AOA. Our results demonstrate that Mn toxicity induces a distinct shift in soil N partitioning, characterized by a marked accumulation of AN and concurrent depletion of NN. This imbalance directly reflects a functional impairment in the nitrification pathway, which was associated with a 44.87%-46.50% reduction in AOB amoA genes under the +Mn treatment. The close link between the AOB decline and nitrification inhibition aligns with the well-established role of AOB as key mediators of ammonia oxidation in agricultural ecosystems (Rütting et al., 2021). In contrast, the relative stability of AOA amoA gene abundance suggests that AOA has a higher intrinsic tolerance to Mn stress, which likely stems from its evolutionary adaptation to acidic niches (Bai et al., 2015). This differential response between AOB and AOA may be further exacerbated by Mn-induced soil acidification, as AOB activity is strongly pH-dependent (Nicol et al., 2008), whereas AOA exhibits greater resilience under low-pH conditions. Under Mn toxicity, the abundance of amoA genes in AOB significantly decreased at both N levels, while the abundance of amoA gene in AOA and the AOA/AOB ratio exhibited downward and upward trends, respectively. However, these differences were not statistically significant. In addition, Mn toxicity significantly reduced the nitrification potential of soils at both N levels, indicating that Mn toxicity inhibits soil nitrification. This finding is consistent with the results of the laboratory incubation in this study, demonstrating that AOB are more sensitive to Mn toxicity and are more significantly inhibited than AOA. Despite the stable abundance of AOA, their inability to compensate for the inhibited AOB suggests limited functional redundancy under Mn stress. This pattern may be related to substrate specificity: AOA prefer low ammonia concentrations (Stopnišek et al., 2010), whereas the high N inputs in this study favored AOB. In low-N or chronically Mn-stressed acidic soils, AOA might contribute more substantially to nitrification. Further correlation analysis between the abundances of AOB and AOA amoA genes and soil physicochemical factors revealed that AOB amoA gene abundance was significantly positively correlated with soil pH and NN content, whereas AOA amoA gene abundance had no significant correlation with these factors. This suggests that soil pH is the primary factor driving the variations in AOB amoA abundance. AOB are more susceptible to changes in soil pH, while AOA exhibit stronger tolerance to low pH and are better adapted to acidic environments. These findings align with those of Nicol et al. (2008) and Bai et al. (2015). Furthermore, while Pang et al. (2025) focused on the regulation of Mn bioavailability by plant secondary metabolites, this study demonstrates the core role of microorganisms, particularly AOB, in mediating Mn toxicity through disruptions in nitrogen cycling. Together, these findings indicate that the effects of Mn toxicity on the sugarcane-soil system arise from multi-interface interactions among plants, microorganisms, and nutrients. This novel, detailed examination of the microbial mechanisms underlying Mn toxicity can inform strategies for mitigating Mn-induced nitrification inhibition in acidic soils.

The results from α-diversity analysis of AOB and AOA indicated that Mn toxicity had no significant effect on the diversity of AOA at either the N level or the diversity of AOB at the N1 level, but significantly increased the diversity of AOB at the N2 level. This indicates that Mn toxicity may partially enhance AOB diversity in the soil under certain N conditions. This phenomenon may be associated with factors influencing the activity of ammonia-oxidizing microorganisms. Previous studies have shown that the activity of these ammonia-oxidizing microorganisms is determined by the concentration of soluble heavy metals rather than the total heavy metal content (Kong et al., 2006; Semerci and Çeçen, 2007). Based on measured available Mn content under the exogenous addition of 328 mg·kg^-1^ Mn, the exchangeable Mn content in the rhizosphere soil of the N1 level Mn toxicity treatment was 321.83 mg·kg^-1^, whereas that of the N2 level was 225.28 mg·kg^-1^. These differences in exchangeable Mn content may be the primary factor underlying the observed variation in AOB community diversity in the rhizosphere soil.

Community structure composition analysis of AOB and AOA in the rhizosphere soil revealed that the dominant phylum for AOB was Proteobacteria, with the dominant genus being Nitrosospira (accounting for 82.9%–99.3% and 71.1%–96.4%, respectively). Numerous studies have shown that AOB in soil are primarily composed of Proteobacteria and Nitrosospira at the phylum and genus levels (Beman et al., 2007; Zhang et al., 2012; Li et al., 2018). For AOA, the species mainly belonged to the *Thaumarchaeota* phylum, consistent with previous findings (Jie et al., 2015). Statistical analysis of the relative abundance of the dominant bacterial groups in each treatment revealed that Mn toxicity significantly reduced the relative abundance of *unclassified_c_Betaproteobacteria* at the genus level of AOB at the N1 level. However, the relative abundance of this bacterial group was very low across all treatments, at only 0.2%–3.1%, indicating that Mn toxicity had a limited effect on the overall AOB community composition. In contrast, for AOA, Mn toxicity significantly increased the relative abundance of Crenarchaeota at the phylum level and Nitrososphaera at the genus level under N2 conditions, suggesting that Mn toxicity may promote their proliferation to some extent.

Numerous studies have shown that the community structure of ammonia-oxidizing microorganisms is closely associated with the fundamental physicochemical properties of the soil. NN, TN, temperature, and nitrite-N are among the four most significant environmental factors influencing the succession and diversity of ammonia-oxidizing archaeal communities (Xie et al., 2016). Dai et al. (2015) reported that AOB communities are closely related to soil TN and SOM contents. Chen et al. (2020) discovered that soil pH, TN, and NN contents have significant impacts on AOA communities, whereas Stopnišek et al. (2010) indicated that changes in soil pH are the primary drivers of AOA community shifts. The redundancy analysis revealed that the physicochemical properties of the rhizosphere soil significantly affected the microbial community structures of AOA and AOB. Among these factors, soil organic carbon content and TN content exerted the greatest influence on AOB, whereas soil pH and NN content had the greatest impact on AOA, consistent with previous research. Correlation analysis also showed that soil nitrification potential, along with the abundances of AOB and AOA amoA genes, were significantly correlated with soil physicochemical properties. Moreover, soil nitrification potential and AOB amoA gene abundance were significantly correlated with N accumulation in sugarcane roots, shoots, and whole plants, as well as with N uptake efficiency. These results suggest that Mn toxicity alters the physicochemical properties of the rhizosphere soil, thereby inhibiting soil nitrification by disrupting ammonia-oxidizing microbial communities.

This study addressed two core questions: first, whether Mn toxicity suppresses nitrification through changes in AOB/AOA communities, and second, whether such changes reduce nitrogen availability for sugarcane. Our results support both hypotheses. Mn toxicity inhibits AOB abundance and nitrification potential, leading to decreased NN levels and reduced nitrogen uptake efficiency in sugarcane. Additionally, the hypothesis that AOB are more sensitive to Mn than AOA was confirmed. This finding helps explain the observed nitrification block in acidic soils, where AOA typically dominates but fails to functionally compensate for the inhibited AOB under the tested conditions.

### 4.2 Effects of Mn toxicity on sugarcane growth, development, N uptake, and N utilization

Through simulated indoor culture and pot experiments, we thoroughly investigated the effects of Mn toxicity on sugarcane growth and development. The results indicated that Mn toxicity significantly inhibited sugarcane growth, primarily manifesting as significant reductions in dry matter accumulation in the roots, shoots, and whole plants, along with a notable decline in plant height. This finding aligns with that of Long (2020), who pointed out that excessive Mn causes leaf chlorosis, stunted growth, and typical Mn toxicity symptoms in sugarcane. This phenomenon likely results from Mn over accumulation in plant tissues, which disrupts physiological metabolism and damages cellular structures. For example, Mn toxicity severely inhibits sugarcane root growth and alters root morphology and physiological functions (Huang et al., 2016). Excessive Mn may also interfere with chlorophyll synthesis, resulting in leaf chlorosis and reduced photosynthetic efficiency (Yang et al., 2022). Furthermore, Mn toxicity can disrupt plant hormone synthesis and transport, ultimately affecting plant growth (Takagi et al., 2021).

The significant positive correlation between N content in sugarcane roots and shoots and the abundance of AOB amoA genes under Mn toxicity further confirms the central role of AOB in regulating sugarcane N utilization. Mechanistically, Mn toxicity significantly impairs soil nitrification by inhibiting the abundance and activity of AOB, leading to the accumulation of AN and a deficiency of NN, the latter being the primary form of N absorbed by sugarcane (Pan et al., 2021). This imbalance in N forms directly restricts the uptake of N by the root system and reduces its translocation to the aboveground parts, ultimately resulting in a decrease in N accumulation in various plant organs. Notably, there was no significant correlation between AOA abundance and sugarcane N content, highlighting that in acidic soils with high N input, AOB rather than AOA are the key microbial functional group driving nitrification and influencing sugarcane N nutrition (Nicol et al., 2008). This finding provided a direction for N management in Mn-contaminated sugarcane fields: protecting AOB communities through measures such as improving soil pH and optimizing N fertilizer forms will be effective approaches to mitigate the harm of Mn toxicity and enhance sugarcane N use efficiency.

Mn toxicity suppresses sugarcane growth and reduces N uptake and utilization efficiency. Specifically, N accumulation in the roots, aboveground tissues, and whole plants decreased significantly, along with a reduction in N uptake efficiency. Mn toxicity alters the physicochemical properties of soil, leading to a significant decrease in NN content and a significant increase in AN content. As sugarcane primarily utilizes NN as its N source, a reduction in available NN in the soil diminishes the plant’s N uptake capacity (Pan et al., 2021). Moreover, Mn toxicity inhibits the growth of the sugarcane root system, reducing the distribution area of the roots in the soil, and consequently lowering their N uptake capacity (Huang et al., 2016). Mn toxicity may also affect the permeability of root cell membranes, further reducing the efficiency of N uptake by roots (Sieprawska et al., 2024).

Furthermore, Mn toxicity inhibited soil nitrification and reduced NN availability in the soil. This study found that under Mn toxicity, the soil nitrification potential and abundance of the AOB amoA gene were significantly reduced. The diversity of AOB significantly increased, with a notable decrease in the relative abundance of *unclassified_c_Betaproteobacteria* at the genus level. Meanwhile, the relative abundances of Thaumarchaeota at the phylum level and Nitrososphaera at the genus level significantly increased. The RDA indicated that soil organic carbon, TN, and AN content were the main factors affecting the AOB community, whereas soil exchangeable Mn, AN content, and soil pH were the main factors influencing the AOA community. Correlation analysis further demonstrated that the soil nitrification potential and abundance of the AOB amoA gene were significantly and positively correlated with N accumulation and uptake efficiency in sugarcane. This further illustrates that Mn toxicity inhibits nitrification, reducing the availability of NN for sugarcane, and thereby lowering sugarcane N uptake and utilization efficiency. This study provides a comprehensive assessment of the interplay among Mn toxicity, soil microbial communities, and plant nitrogen uptake, providing a mechanistic understanding that can guide the development of sustainable agricultural practices for Mn-contaminated acidic soils.

Although this study provided mechanistic insights into the responses of AOA and AOB to Mn toxicity, the analysis was limited to ammonia-oxidizing microorganisms. A more comprehensive metagenomic approach would offer a holistic view of the entire microbial community, encompassing other key functional groups involved in N cycling. Such an approach could reveal broader microbial interactions and functional redundancies that may influence nitrification under Mn toxicities. Future studies employing shotgun metagenomics or transcriptomics are warranted to fully elucidate the microbial mechanisms underlying Mn toxicity disruptions in soil N transformations.

In conclusion, this study clarified that Mn toxicity in sugarcane rhizosphere soil primarily inhibits soil nitrification and impairs sugarcane N uptake by reducing AOB abundance, with soil organic carbon, TN, and exchangeable Mn being the key drivers of AOB/AOA community shifts. However, the limitations of this research should be acknowledged when interpreting its conclusions: a narrow focus on ammonia-oxidizing microorganisms, lack of long-term field verification, insufficient functional/transcriptional data, and reliance on a single soil type and sugarcane variety when interpreting its conclusions. Nonetheless, the findings provide targeted insights for sugarcane management in Mn-contaminated acidic soils: prioritizing nitrate-based fertilizers or slow-release urea to bypass inhibited nitrification, applying lime (with organic amendments) to reduce Mn bioavailability and protect AOB, leveraging intercropping (e.g., sugarcane-peanut) to improve soil health, and monitoring soil exchangeable Mn and AOB abundance for proactive management. Future research addressing the identified limitations (e.g., integrating metagenomics/metatranscriptomics and conducting multi-soil/variety field trials) will further refine our understanding of Mn-N-microbial interactions and optimize sustainable management strategies for sugarcane production in Mn-contaminated areas.

## 5 Conclusion

Mn toxicity significantly decreased both the nitrification potential and AOB amoA gene abundance in sugarcane rhizosphere soil, while also altering the community structures of both AOB and AOA. Soil organic carbon, TN, and AN contents were identified as potential factors influencing the AOB community. In contrast, exchangeable Mn, AN, and soil pH were the main factors affecting the AOA community, and nitrification potential and AOB amoA gene abundance showed significant positive correlations with sugarcane N accumulation and uptake efficiency. In conclusion, Mn toxicity affected AOB and AOA communities in the sugarcane rhizosphere, thereby inhibiting soil nitrification. This ultimately reduces the availability of nitrate-N in sugarcane, leading to decreased crop N uptake and utilization efficiency.

## Data Availability

The raw sequencing data for both the metagenomic datasets and the 16S rRNA amplicon datasets utilized in this study are available at the China National Gene Bank under the accession number PRJNA1237512.
